# An Unusual Cause of Failure to Ventilate

**DOI:** 10.1177/2324709618781174

**Published:** 2018-06-13

**Authors:** John T. Denny, Sagar S. Mungekar, Benjamin R. Landgraf, Zoe M. Rocke, Valerie A. McRae, Christian P. McDonough, James T. Tse, Scott J. Mellender, Geza K. Kiss

**Affiliations:** 1 Rutgers University, New Brunswick, NJ, USA; 2 St. George’s University, Grenada, West Indies

**Keywords:** ventilator complications, failure to ventilate, ICU equipment malfunction, complication of intubation, endotracheal tube malfunction

## Abstract

We report an unusual case of endotracheal tube failure. It was due to a manufacturing defect in the internal white plastic piece that is normally depressed by the luer-lock syringe within the blue pilot balloon. Prior to use, the endotracheal tube was tested and functioned normally. A 64-year-old patient in the intensive care unit with a history of hypertension was being mechanically ventilated after uneventful abdominal surgery. After several hours in the intensive care unit, he was noted to be suddenly no longer receiving adequate tidal volumes from the ventilator. It was found that the cuff on the endotracheal tube was not retaining air when it was filled with air from a syringe. This lead to a large “leak” around the endotracheal tube such that the intended tidal volumes set on the ventilator were not delivered to the patient. The patient was uneventfully reintubated and did well. Subsequent investigation revealed the cause to be a manufacturing defect in the internal white plastic piece that is normally depressed by the luer-lock syringe within the blue pilot balloon. Other mechanisms of cuff failure are reviewed in this case report. This case is an unusual reason for cuff failure. Illustrations supplied alert the reader how to identify the appearance of this manufacturing defect in a pilot balloon. This case illustrates the potential device malfunctions that can develop during a procedure, even when the equipment has been tested and previously functioned well. Even small defects developing in well-engineered products can lead to critical patient care emergencies.

## Background

**Figure 1.**
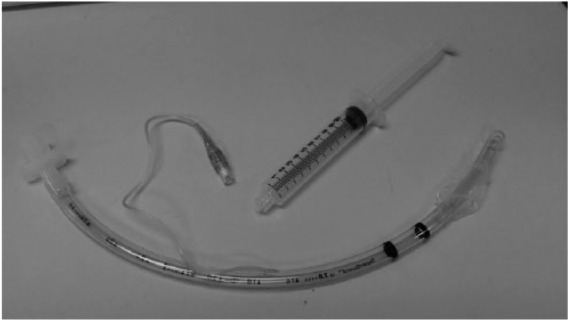
A typical endotracheal tube with attached pilot balloon.

Endotracheal tubes are used to provide mechanical ventilation while the patient is under general anesthesia. Cuffed endotracheal tubes have an inflatable balloon at the distal end providing a significant advantage over noncuffed varieties; they allow for higher positive pressure during ventilation and better secure the airway decreasing the risk of aspiration^1^ (Figure 1). Aspiration refers to the passage of either oropharyngeal secretions or gastric contents into the trachea.

**Figure 2.**
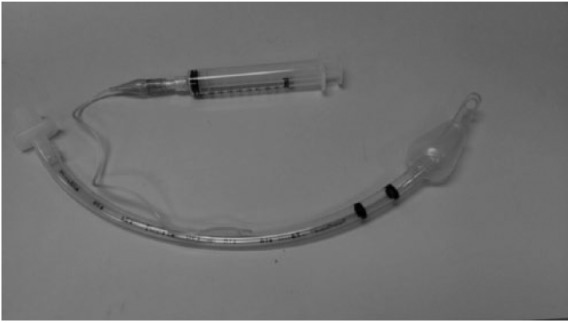
Endotracheal tube with syringe inflating cuff on the distal end of the endotracheal tube.

After the patient is anesthetized, a laryngoscope is used to enhance the oral pharyngeal view, ultimately showing the patient’s glottic opening to the “vocal cords.” The endotracheal tube is then carefully inserted through the oral pharynx and the glottis into the trachea. The cuffed balloon is gently inflated with air using a luer-lock syringe attached to the blue pilot balloon. A valve just inside the luer connection keeps the air trapped inside the inflated cuff (Figure 2).

The tube position is then verified through capnography and auscultation.

## Case Presentation

**Figure 3.**
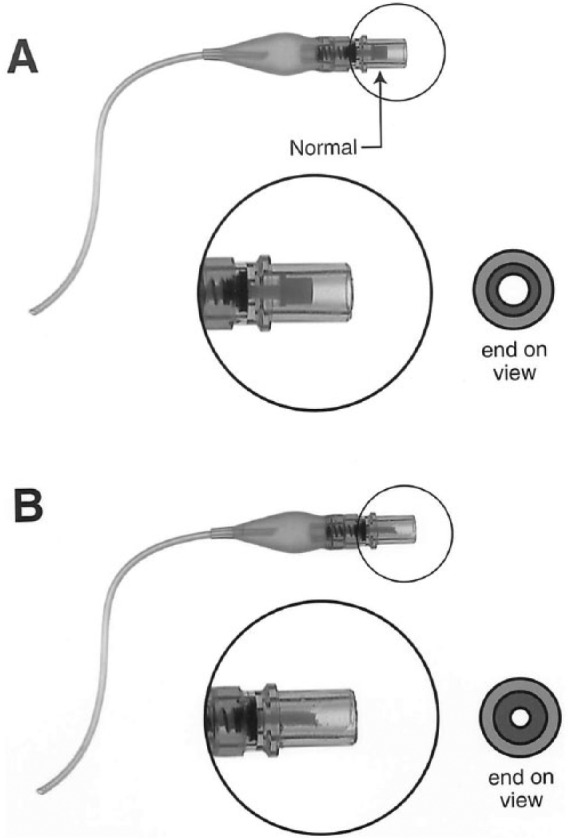
(A) The normal appearance of the blue pilot balloon. (B) The manufacturing defect that caused the loss of cuff volumes out, leading to a leak and reduction in ventilator volumes. The altered appearance of the smaller diameter white plastic piece can be seen especially in the cross-sectional view of the end of the blue pilot balloon.

We report an unusual case of endotracheal tube failure due to a defective and malfunctioning valve apparatus inside the blue pilot balloon. A 64-year-old patient with a history only of hypertension had undergone uneventful abdominal surgery. As is the usual practice at our institution, the equipment was tested prior to use. Specifically, the laryngoscope and the cuff on the endotracheal tube were tested. The cuff on the laryngoscope was tested by attaching a 10 cc luer-lock syringe, inflating the cuff, disconnecting the syringe, evaluating the cuff for integrity and retention of air. The cuff was then deflated. The intraoperative course was uneventful; set tidal volumes were delivered, and there was no evidence of a cuff leak. Postoperatively, the patient was being ventilated uneventfully in the intensive care unit (ICU) after surgery. No ventilator alarms were alarming. Two hours later, it was noted that he was no longer receiving adequate tidal volumes from the ventilator. On further investigation, the cuff on the endotracheal tube was not retaining air when it was filled with air from a syringe. This led to a large “leak” around the endotracheal tube such that the intended tidal volumes set on the ventilator were not being successfully delivered to the patient. The patient was uneventfully reintubated and did well thereafter. Subsequent investigation revealed the cause to be a manufacturing defect in the internal white plastic piece that is normally depressed by the luer-lock syringe within the blue pilot balloon. This is illustrated in Figure 3A and B.

The endotracheal tube and cuff passed inspection prior to use, and functioned well for a period of hours. Thus, any defect developed suddenly many hours after inspection and use. It is hypothesized that there was a manufacturing defect resulting in a fracture of the internal white plastic piece that is normally depressed by the luer-lock syringe within the blue pilot balloon. This presumably resulted in a loss of cuff pressure and the resulting low tidal volume alarms on the ventilator. The fracture of the white plastic piece then also produced the altered appearance seen in Figure 3B, of the smaller diameter white plastic piece. It is unknown, but possible, that there was a manipulation of the cuff with a syringe after hours in the ICU that then led to the fracture of the white plastic piece, and the cuff failure.

As with all equipment, device problems or failure can occur. With the endotracheal tube, such problems often involve the pilot balloon and cuff assembly. This subassembly includes the blue pilot balloon, the thin tubing running from the blue pilot balloon along the edge of the endotracheal tube, and the inflatable cuff itself. The pilot balloon can fail or leak.^2^ Within the blue pilot balloon is a spring-loaded valve. When a luer-lock type syringe is screwed into the female end, the syringe tip contacts the white plastic piece and depresses the spring-loaded valve and allows for air passage to or from the distal cuff. Normally, when the luer-lock syringe tip is removed from the blue pilot balloon connector, the spring-loaded valve will automatically close, preventing any air from escaping the cuff. The thin tubing that runs along the endotracheal tube to the distal cuff can be broken or damaged. This can occur during patient care activities in the ICU, such as shaving the patient. It can also occur in an agitated patient who may bite through the thin tubing. This can usually be prevented through careful sedation of the patient. The use of a soft bite block can also be helpful in preventing the patient’s teeth from impinging on the endotracheal tube or the thin tubing of the pilot balloon. A soft bite block can easily be made by rolling several 4 × 4 inch gauze sponges and then wrapping tape around them circumferentially. Manufacturing defects involving the attachment of the thin tubing to the endotracheal tube have also been reported.^3,4^

The inflatable cuff at the distal end of the endotracheal tube can also fail. It may incur damage on the initial insertion via unnoticed abrasion against sharp teeth. If a significant tear occurs, the cuff failure will be immediately evident through failure to deliver adequate tidal volumes. However, very subtle damage to the cuff may result in delayed presentation many minutes after the original intubation. Alternatively, the cuff may fail later from use or overinflation.

## Checklists

Checklists are sometimes used to facilitate consistently performing steps of a complicated procedure.^5^ Checklists have been described across varied disciplines and procedures including neonatal,^6^ interventional radiology,^7^ and paracenteces.^8^ Smith et al showed that using a preintubation checklist for emergency department intubation of trauma patients was associated with a decrease in intubation-related complications, decreased length of time from paralysis-to-intubation, and better compliance with recognized safety measures.^9^ Especially for non–anesthesia providers, checklists are a helpful asset prior to intubation. There is variability in checklists among institutions.^10,11^

This case illustrates the potential device malfunctions that can develop during a procedure, even when the equipment has been tested and previously functioned well. Even small defects, such as the manufacturing defect leading to cuff failure described here, that develop in well-engineered products can lead to critical patient care emergencies. This case report shows an unusual reason for cuff failure. The illustrations supplied allow for visual identification of the appearance of a manufacturing defect leading to valve failure in a pilot balloon.
